# Ibuprofen - a Safe Analgesic During Cardiac Surgery Recovery? A Randomized Controlled Trial

**DOI:** 10.15171/jcvtr.2015.31

**Published:** 2015-11-26

**Authors:** Saddiq Mohammad Qazi, Eske Jesper Sindby, Martin Agge Nørgaard

**Affiliations:** Department of Cardiothoracic Surgery, Aalborg University Hospital, Hobrovej 18-22 9000 Aalborg, Denmark

**Keywords:** Cardiac Surgery, Non-steroidal Anti-inflamatory Drugs, Acute Renal Injury, Analgesia

## Abstract

***Introduction:*** Postoperative pain-management with non-steroid anti-inflammatory drugs has been controversial, due to related side-effects. We investigated whether there was a significant difference between an oxycodone-based pain-management regimen versus a slow-release ibuprofen based regimen, in a short term post-cardiac surgery setting. Particular attention was given to the rate of myocardial infarction, sternal healing, gastro-intestinal complications, renal failure and all-cause mortality.

***Methods:*** This was a single-centre, open label parallel design randomised controlled study. Patients, who were undergoing cardiac surgery for the first time, were randomly allocated either to a regimen of slow-release oxycodone (10 mg twice daily) or slow-release ibuprofen (800 mg twice daily) combined with lansoprazole. Data relating to blood-tests, angiographies, surgical details and administered medicine were obtained from patient records. The follow-up period was 1 to 37 months (median 25 months).

***Results:*** One hundred eighty-two patients were included in the trial and available for intention to treat analysis. There were no significant difference between the groups (P>0.05) in the rates of sternal healing, postoperative myocardial infarction or gastrointestinal bleeding. The preoperative levels of creatinine were found to increase by 100% in nine patients (9.6%) in the ibuprofen group, resulting in an acute renal injury (in accordance with the RIFLE-criteria). Eight of these patients returned to normal renal function within 14 days. The levels of creatinine in patients in the oxycodone group were not found to increase to the same magnitude.

***Conclusion:*** The results of this study suggest that patients treated postoperatively, following cardiac surgery, are at no greater risk of harm if short term slow release ibuprofen combined with lansoprazole treatment is used when compared to an oxycodone based regimen. Renal function should, however, be closely monitored and in the event of any decrease in renal function ibuprofen must be discontinued.

## Introduction

Postoperative pain following median sternotomy can represent a major postoperative problem as it can lead to reduced mobilisation, shallow, restricted breathing and insufficient cough, which can lead to pulmonary complications.^1^

Pain management with opioids has frequent side-effects such as confusion, respiratory depression, sedation, nausea and obstipation. Non-steroid anti-inflammatory drugs (NSAIDs) can be used as opioid-sparing analgesics following cardiac surgery. However, this has recently become controversial, as NSAIDs have been linked to an increased risk of acute myocardial infarction, especially in patients with ischaemic heart disease, as well as renal failure, gastrointestinal (GI) bleeding and impaired bone healing.^2-5^

The purpose of this study was to investigate whether the side-effects of short term ibuprofen treatment occurred at an increased rate, when compared with a standard oxycodone based regimen. The study was randomised controlled trial of patined.

## Materials and Methods

### Trial Design

Between 1st April 2009 to 31st December 2010, we conducted a single-centre, open label, parallel designed randomised controlled trial at the Department of Cardio-Thoracic Surgery, Aalborg University Hospital, Denmark. The aim of this study was to assess the effects of an ibuprofen based regimen, on an “as needed” opioid usage, following cardiac surgery.

The patients were initially randomised 1:1 to either receiving slow-release oxycodone (10 mg twice daily) or slow-release ibuprofen (800 mg twice daily) combined with lansoprazole. Patients were randomised using consecutively numbered randomisation in sealed and tamper-proofed envelopes.

The study was sufficiently powered at 80% with a significance level of 5%, in order to detect a difference of 5 micromol/l in creatinine between the means of 80 micromol/l and 85 micromol/l, with a standard deviation of 10 micromol/l.

Data was entered into a database prospectively, and internally audited for missing and erroneous data by the lead investigator.

At follow up, data regarding myocardial infarction, renal function, sternal healing, gastric bleeding and all cause mortality were retrieved from the medical records of all patients.

### Population

We included patients undergoing first-time elective cardiac surgery including full median sternotomy from 1st April 2009 to the 31st December 2010. The surgical procedures included on- and off-pump bypass surgery, aortic and mitral valve substitutions and repairs, maze and mini-maze operations. Combinations of these procedures are also included. The inclusion criteria included patients aged >18 years undergoing full median sternotomy for the first time. The preoperative exclusion criteria were other forms of sternotomy (i.e., re-sternotomy or partial sternotomy) preoperative creatinine above 110 µmol/L, preoperative use of opioids or NSAIDs in analgesic doses (aspirin in antithrombotic doses was accepted), allergy to NSAIDs or opioids, and other contraindications to the used drugs, including a history of GI-ulcers. The postoperative exclusion criteria were a stay of more than one night in the intensive care unit (ICU), unacceptable side effects, exclusion at the patients request, and insufficient pain-relief (evaluated during ward rounds twice-daily). Patients remained in their respective group allocation if either ibuprofen or morphine treatment was ceased, however an “intention to treat” analysis was applied to these cases.

**Figure 1:**
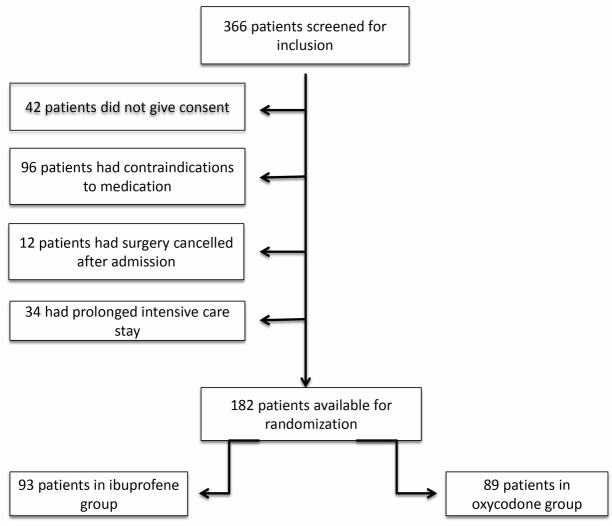
Inclusion & Exclusion.

Out of a total of 366 patients screened, 182 patients fulfilled our inclusion criteria and consented to be included in the study (Figure 1).

### Intervention

In the ibuprofen regimen, slow-release ibuprofen (“Brufen Retard”, Abbott, Copenhagen, Denmark, 800 mg two times daily) was the basic analgesic.

The opioid regimen included a basic dose of slow-release oxycodone (“Oxycontin”, Norpharma, Vedbæk, Denmark, 10 mg two times daily).

For both regimens paracetamol 1g x 4 was administered routinely, and oxycodone as injection (2.5 mg i.v.) or capsule (5 mg) was available for break-through pain. This was administered on a “as needed” basis (PRN). Lansoprazol (“Lansoprazole”, Actavis, Gentofte, Denmark, 30 mg once daily) was co-administered to prevent development of gastric ulcers in both groups. Furthermore magnesiumoxide (1g two times daily) and sodiumpicosulfate (10 mg once daily) were co-administered to prevent obstipation in all patients.

These regimens were initiated on the first postoperative day, when the patient was transferred from the ICU to the ward, and continued until the seventh day, postoperatively. Some patients continued the pain management regime after transfer to other departments, or discharge from the hospital. Pain management was, thereafter, the responsibility of either the receiving department or the patient’s general practitioner, although not as part of the protocol.

### Data collection

Baseline data were recorded at patient inclusion. Surgical procedure details, biochemistry and postoperative complications were retrieved from the electronic patient records. Even though the patients only underwent a medical intervention for a short period, the follow-up period extended to a median of 25 months (1-37 months) after surgery. An extensive follow-up period was intentional, in order to be able to identify any long term effects to this short term treatment. Information relating to any subsequent complications and the time at which these occurred was retrieved from the electronic patient records either during the hospitalisation, any subsequent re-admissions, or out-patient contacts. All-cause mortality was assessed through the national Civil Registration System database. As the electronic patient record is nationwide, the follow-up was 100%.

### Outcome measures

The following postoperative ibuprofen-related complications were identified:

- Sternal non-union, defined as a loose sternum more than one month after surgery requiring re-operation. The one month limit was used to exclude other immediate surgical causes for re-opening the sternum.
- Postoperative myocardial infarction according to ESC/ACC/AHA/WHF consensus-report during the follow-up period.^6^
- Gastrointestinal bleeding (clinical symptoms leading to gastroscopy and/or colonoscopy) during the follow-up period.
- Renal injury according to the RIFLE-criteria (measuring s-creatinine from preoperatively until discharge (at least on day 1, 2 and 4), and also at follow-up).
- All-cause mortality during the follow-up period.

The RIFLE-criteria were developed by The Acute Dialysis Quality Initiative (ADQI) in need of consensus and/or evidence-based guidelines for the treatment and prevention of acute renal failure.^7^ The RIFLE criteria stage define acute renal injury as a progressive disease ranging from “Risk, Injury, Failure, Loss “to “End stage” renal disease. Staging is made possible by measuring an increase or decrease in the levels of s-creatinine, relative to a baseline measurement (in the case of this study a preoperative measurement). The sensitivity for acute renal injury decreases through the classification, whereas the specificity increases. The staging system has been validated for cardiac patients and a higher RIFLE-stage was correlated to higher mortality, after cardiac surgery.^8^ Furthermore, Lassnig et al found that even minimal changes in postoperative creatinine affected the 30 day mortality following cardiac surgery.^9^

Information regarding all cause mortality was retrieved using the electronic patient management system for Northern Jutland, which is synchronised with the national Civil Registration System database, including vital status.

### Statistics

Data are presented as number of patients, medians, and ranges. Patients were compared according to their group allocation, i.e. either ibuprofen, or oxycodone group and statistical calculations were performed using an “intention-to-treat”-design.

Fischer’s exact test, Mann-Whitney, and 95% confidence intervals were used as appropriate. Significance was defined as P values below 0.05.

## Results

During the 1 year 8 month inclusion period 182 patients were included in the trial. Of those, 93 patients were randomised to the ibuprofen-group and 89 patients were randomised to the oxycodone group.

Thirteen patients in the oxycodone group were excluded, due to insufficient pain-relief or adverse side effects, as a result of receiving oxycodone. These 13 patients received the alternative of either supplemental ibuprofen or ibuprofen only.

Five patients in the ibuprofen group were excluded due to side effects. However, this did not influence the results as the analysis was performed using “intention to treat”-design. The median ibuprofen treatment duration during hospital stay was five days.

As seen in Table 1, the groups appeared to be comparable regarding demographic- and procedural data.

**Table 1:** Demographics and Procedures

	**Ibuprofen (n = 93)**	**Opiod (n = 89)**	*** P *** **Value**
Patient demographics			
Age (range‏)	67‏ (37-84‏)	66‏ (29-87‏)	0.67
Gender (male/female‏)	69/24	73/16	0.27
BMI (mean, +/- SD‏)	27‏ +/- 4.1	27‏ +/- 4.6	NS
Risk factors			
Hypertension	65	59	0.64
Hyperlipidaemia	65	66	0.63
Diabetes	20	16	0.58
COPD	9	9	1.00
Perifereal artery disease	8	5	0.57
Previous stroke	5	3	0.72
EF <30%	2	2	1.00
EF 30%-50%	29	28	1.00
EF >50%	62	58	0.87
Euroscore (mean +/- SD‏)	4.49‏ +/- 2.66	4.67‏ +/- 2.89	NS
Procedures			
Cross-clamp time (minutes +/- SD‏)	68‏ (+/- 31‏)	60‏ (+/- 27‏)	0.18
Lowest core temperature (C° +/- SD‏)	35.5‏ (+/- 0.8‏)	35.5‏ (+/- 0.9‏)	0.91
Induced hypothermia, No	13	6	0.22
Bypass procedures	35	47	0.23
OPCAB	12	6	0.22
Valve procedures	28	20	0.31
Combo procedures	14	14	1.00
Other procedures	4	2	0.68

NS: Non-significant‏.

With regards to bone healing and postoperative myocardial infarctions, there were no significant differences between the groups (P > 0.05) (Table 2). There was a non-significant higher rate of gastrointestinal bleeding in the ibuprofen-group, when compared to the oxycodone group not receiving supplemental ibuprofen at any time (five versus two respectively). The GI bleeding in the ibuprofen group occurred 1, 7, 29, 41, and 53 weeks post-operatively. The bleeding occurring 1 week after surgery was in a patient with fresh blood in her stools. A source of the bleed could not be located during a subsquent colonoscopy, however, the bleeding ceased without intervention and gastroscopy was not performed.

**Table 2:** Complications

**Complications**	**Ibuprofene** **(n=93)**	**Opiod** **(n=89)**	**P-values**
Acute myocaridal infarction	1	0	1.00
Sternal nonunion no.	0	0	1.00
Gastrointestinal bleeding	5	2	0.44
RIFLE risk	9	3	0.14
RIFLE injury	9	0	0.01*
RIFLE failure	0	0	1.00
RIFLE loss	0	0	1.00
RIFLE end stage	0	0	1.00
30 day mortality	1	1	1.00
Overall mortality	3	5	0.49
Change of medical regimen	5	13	0.04*

**Figure 2:**
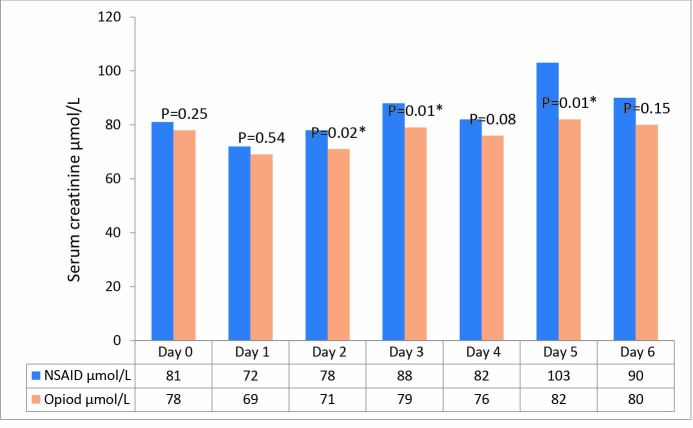
Serum Creatinine Development.

**Figure 3:**
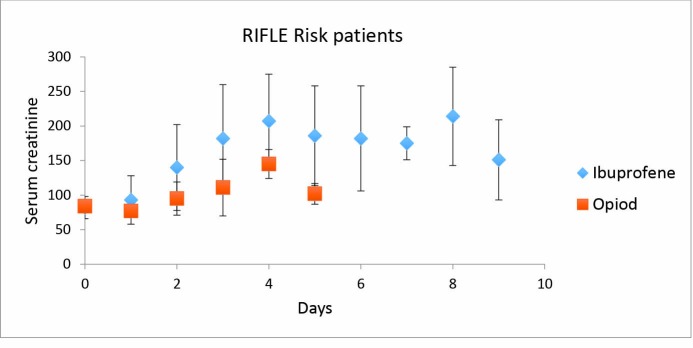
RIFLE Risk Patients - Serum Creatinine Development

We found a significantly higher se-creatinine in the ibuprofen group on days two, three and five post-operatively (Figure 2). The levels of creatinine increased in nine patients in the ibuprofen group, compared to three in the oxycodone group, this increase in creatinine was by >50% above the preoperative level within the first 6 days after surgery (Figure 3), corresponding to “Risk” in the RIFLE classification. A further nine patients in the ibuprofen group experienced a doubling of their preoperative creatinine, corresponding to an acute renal “Injury” in the RIFLE classification. These nine patients all returned to values below the double preoperative creatinine level within 5-13 (mean 9) days after surgery, and all but one had normalised at follow-up. The patient that did not normalise his renal function had a marginally increased serum creatinine of 119 µmol/L on final follow-up. None of the patients required dialysis of any form.

In the oxycodone group, three patients increased their creatinine by >50% above the preoperative values. None of the patients in the opioid group doubled their creatinine.

There were two mortalities within the first 30 days after surgery. A 66-year old man, who was randomised to the ibuprofen group, suffered a postoperative AMI due to a left internal mammary artery anastomosis and subsequent left ventricular failure. He was treated with a left-sided trans-femoral intra-aortic balloon pump. Unfortunately, the left leg became ischemic and multiple organ failure followed. Eight days following the procedure, the patient died as a result. The second mortality was a 63-year old woman randomised to the oxycodone-group. She was unable to be revascularised completely during CABG-surgery (oronary artery bypass graft) and sudden cardiac death ensued seven days after surgery, after transfer to another hospital.

There was no difference in all-cause mortality between the groups (follow-up: 2.1 years, range 0.01-3.16 years.

## Discussion

Ibuprofen is a non-selective reversible inhibitor of the cyclooxygenase (COX) enzyme, responsible for converting arachidonic acid into prostaglandin H_2_, which is a precursor for prostaglandin E_2_, which can induce fever and inflammation. As described below, the inhibition of prostaglandin synthesis can be associated with a number of potential side-effects because prostaglandins are involved in a number of physiological processes.

Our results do not point towards an increased risk of myocardial infarction, impaired bone healing, GI-bleeding, or all-cause mortality when using ibuprofen in selected patients during cardiac surgery recovery. An increase in levels of serum creatinine was, however, observed in some patients.

These possible disadvantages have to be weighed against the benefits of an opioid-sparing pain management regime, including less opioid side-effects.^10^ Furthermore, an added analgesic effect and a lower Visual Analogue Score (VAS) has previously been reported when using NSAID-based pain regimens.^1,11,12^

###  Myocardial Infarction

In our study a single patient from the ibuprofen group suffered a postoperative myocardial infarction caused by an anastomotic problem, as described above.

An increased risk of AMI during NSAID-treatment has been suggested to be caused by an inhibition of COX-2 in the endothelium, responsible for the production of prostacycline, which inhibits platelet aggregation and is a vasodilator.^13^ Furthermore, COX-1 is only affected to a lesser extent and the production of thromboxane, responsible for platelet aggregation, is not suppressed equally. This could also explain why COX-2 selective NSAIDs are more thrombogenic. However, a direct relationship between COX-selectivity and the risk of myocardial infarction has not been firmly documented.^14^

In Schjerning et al retrospective cohort study, including 35 257 patients with a medical record with previous myocardial infarction, even a short term treatment with NSAID posed an increased risk with an odds ratio of 1.45 (1.29-1.62).^2^ However, the use of ibuprofen for less than a week had an odds ratio of only 1.04 (0.83-1.30), which the authors found to be insignificant. Roumie et al conducted a retrospective cohort study with 610 001 patients (14% with cardiovascular disease) and did not find ibuprofen to increase the risk of cardiovascular events (i.e., AMI, ischaemia, CV-death) in patients with CVD (cardio-vascular disease).^15^ In these large out-of-hospital studies, short term ibuprofen treatment does not seem to pose a risk for patients with cardiovascular diseases.

In the postoperative setting after cardiothoracic surgery, one study has found COX-2 selective drugs to increase the risk of postoperative AMI.^16^ However, a meta analysis, including 1065 patients from 20 randomised trials, including both cardiac and thoracic surgery did not find non-selective NSAIDs to increase the risk of myocardial infarction.^10^

Patients with existing coronary disease included in our study underwent bypass-surgery, prior to ibuprofen administration. They were expected to be fully revascularised after this, unlike the cohorts of non-surgical patients reported in other studies.

The results of both previous publications and our study have found the post-operative short term treatment with ibuprofen to be safe with regards to myocardial infarction.

### Bone Healing

Due to the supposed role of prostaglandins in the bone healing process, NSAIDs have been suspected to impair the bone healing process. This has, to some extent, been shown in animal models but clinical evidence in humans has been dubious.^17,18^ A systematic review found that the higher the quality of the study, the weaker the link between impaired bone healing and NSAIDs.^19^ In our study, we did not find any patients with sternal non-union in either group.

### Gastrointestinal Complications

Gastro-intestinal bleedings is a general concern associated with the use of NSAIDs, especially after surgery when the gastric mucosa is more vulnerable due to peroperative ischaemia and the surgical stress-response.^20^ This is due to the inhibition of COX-1 in the gastric mucosa, where prostaglandins are involved in bicarbonate and mucous production. However, the GI-risk associated with the short term use of ibuprofen after surgery is not clear.^21^

One of our patients had fresh blood in her stools, 1 week after the cardiac procedure. The source of the bleeding was not located and the bleeding ceased without intervention. The other identified gastrointestinal bleedings appeared quite late, up to 1 year after surgery and were unlikely to be caused by the short term administration of ibuprofen postoperatively. The prophylatic use of lansoprazole may have contributed to lowering the number of gastrio-duodenal bleeding, as shown in a retrospective study by Fan et al. including 6316 CABG patients, where prophylactic omeprazole lowered the incidence of GI-bleeding (OR=0.19).^22^

### Renal Complications

The peri- and postoperative use of NSAIDs in non-cardiac surgery has been shown to affect the renal function to a limited and clinically non-significant degree in healthy adults.^23^ However there are a number of factors, relating specifically to cardiac surgery, that might make this population more vulnerable to the effects of NSAIDs.

The risk of renal injury during cardio-pulmonary bypass (CBP) is well-documented and in part mediated by renal ischaemia and systemic inflammation.^24^ The renal injury is initiated by medullary hypoxia that arises during CBP.^25^ This can lead to ischaemic endothelial cell dysfunction, with a decline in glomerular filtration rate and disruption of normal nephron histology, such as endothelial cell exfoliation and tubular obstruction. At the same time inflammatory cytokines, such as TNF-alfa, IL-1 and IL-8, are released and facilitate neutrophile adhesion to the endothelium and can initiate an inflammatory cascade.^26^ Other effects of CBP are decreased levels of the vasodilator nitric oxide, hemolysis with release of free haemoglobin, oxidative stress from free iron, and finally renal reperfusion which also produces oxidative stress and further injury to the tissue.^27,28^ Although the initial ischemic insult is short, the inflammatory processes has been initiated, and the decline in GFR lasts much longer than the period of ischaemia.^26^

We choose to include patients undergoing off-pump coronary artery bypass, even though these patients theoretically might have been exposed to a lesser degree of inflammation.^29^ However it is unclear whether this theoretical difference in inflammation, actually affects the degree of renal damage. Schopka et al found no detrimental effect of cardiopulmonary bypass on renal function.^30^ The Coronary Artery Bypass Grafting Surgery Off- or On-pump Revascularisation Study found a short term benefit in favour of OPCAB, however there was no difference in renal function between on- and off pump CABG at 1 year.^31^

The decline in glomerular filtration rate seen during NSAID treatment is thought to be mediated by inhibition of prostaglandin synthesis. Prostaglandins only play a small role in the renal function of healthy adults. In the presence of intracellular dehydration and heart failure the vasodilatory effects of prostaglandins will to some extent, however, counteract the effects of hypoperfusion.^20^ Prostaglandins cause vasodilation of the afferent glomerulus arteriole, an action inhibited by NSAIDs that reduces blood flow to the glomerulus and the glomerular filtration pressure.^32^ This may induce ischaemic damage to the nephrons.

Based on seven randomised controlled trials, one meta-analysis and three retrospective studies Acharya et al did not find NSAIDs in cardiac surgery to be harmful to the kidneys when used early in the postoperative setting.^33^ This review was based on selected patients with no prior renal dysfunction and no contraindications. Our study, however, went further than some of these studies and continued ibuprofen administration for seven days post-operatively. Even though the hemodynamic effects of NSAIDs on the kidney can be found within hours, a prolonged administration may worsen tubular function.20 This could explain our higher proportion of renal impairment (19% in the ibuprofen group, compared with 7.7% otherwise found by Manganic et al following cardiac surgery.^34^ In a recent study by Rafiq et al a multimodal analgesic regimen with ibuprofen was tested, and they found a non-significant tendency towards higher se-creatinine level in the group receiving ibuprofen.^35^

Our findings indicate an increased risk of renal impairment using an ibuprofen-based regimen than when compared to using an oxycodone-based regimen, as seen in the higher proportion of patients doubling their levels of creatinine. The rise in serum-creatinine is seen within two or three days after surgery, and could indicate that renal impairment occurs prior to this because of the time taken for se-creatinin accumulation. The results of this study could suggest that ibuprofen acts as an aggravating factor in the early postoperative treatment, adding to the multifactorial genesis of renal failure.

We speculate whether a reduced dose of slow-release ibuprofen might achieve identical pain-relief whilst reducing renal complications and, furthermore, whether or not reducing the period of ibuprofen treatment from seven to five days would have any negative implications on pain management.

### Strengths and limitations

The strength of this study is primarily the randomization of a selected group of patients undergoing elective surgery, and the length and completeness of follow-up. The period of treatment was sometimes longer than planned in the protocol. This was beyond our control and may have influenced results, by increasing the risk of side-effects.

The limitations of the study includes the limited number of patients, the fact that the intervention with ibuprofen and lansoprazole was only tested against patients receiving oxycodone and not a third groups of patients receiving a ‘placebo’ drug. However, given the nature of the surgical intervention and the fact that the postoperative pain associated with sternotomy does require medical pain management, this was not possible. Finally, in the light of the known risk of gastrointestinal bleeding associated with NSAID, it was deemed unethical to omit lansoprazole for scientific purposes.

## Conclusion

Compared to an oxycodone based pain management regimen, we found it no more hazardous to use slow-release ibuprofen (800 mg twice daily) combined with lansoprazole (40 mg daily) for seven days following cardiac surgery in a selected group of patients, in relation to possible adverse side effects such as myocardial infarction, sternal healing, gastrointestinal bleeding, renal function, and all cause mortality. However, renal function in patients must be closely monitored.

## Acknowledgments

We wish to thank all the surgeons, and nurses who were involved in this study, especially Project Nurse Anita Tracey.

## Ethical issues

All participants gave written consent prior to randomization, upon receiving comprehensive written and verbal information. The study was approved by the local ethical committee for Northern Jutland, Denmark, approval number N20080037. The study was registered at http://clinicaltrials.gov. Registration number: NCT02479165.

## Competing interests

Authors declare no conflict of interest in this study.
